# Editorial: Role of microbial biofilm in infections

**DOI:** 10.3389/fcimb.2023.1231607

**Published:** 2023-06-19

**Authors:** Natalia V. Kirienko, Yuanpu Peter Di

**Affiliations:** ^1^ Department of BioSciences, Rice University, Houston, TX, United States; ^2^ Department of Environmental and Occupational Health, University of Pittsburgh, Pittsburgh, PA, United States

**Keywords:** biofilm, bacterial infections, *C. difficile*, *P. aeruginosa*, anti-biofilm activity

Bacterial infections are a critical concern for health agencies as the rise of antimicrobial resistance continues unchecked across the globe. A key determinant of the infection outcome is the production of biofilms, structured communities comprised of single or multiple species that are engaged in waves of cooperation and competition to obtain the materials they need for growth and division. Intra- and interspecies interactions take place within matrices of extracellular materials, such as DNA, polysaccharides, lipids, and polypeptides, as well as smaller molecules that serve as messengers between and amongst the bacterial species in the community (Ryder et al., 2007; Campoccia et al., 2021; Wong et al., 2021; Katharios-Lanwermeyer and O’Toole, 2022).

Biofilms are thought to have evolved for several reasons, including improved attachment to growth substrates and resisting physical displacement from promising habitats. At some point, bacteria expanded this use of biofilms for increased virulence by preventing phagocytosis, limiting antibody-mediated opsonization, and facilitating colonization of host organisms (Jensen et al., 1990; Leid et al., 2005; Hoiby et al., 2010; Oliver et al., 2019). Additionally, biofilm-related growth is associated with metabolic changes in bacteria, resulting in the emergence of more slowly growing cells with increased antimicrobial resistance (Costerton et al., 1999; Conlon et al., 2015; Tran et al., 2023).

This Research Topic presents a collection of six papers highlighting the roles of biofilms in different aspects of infection. Benyoussef et al. provide a fascinating investigation of the role of motility during biofilm formation and examine what effect this has on bacterial fitness. Specifically, they found that non-motile *E. coli* forms more robust biofilms and produces biofilm more rapidly. Previous research on *Pseudomonas aeruginosa*, coming from George O’Toole, showed that motility genes, including those responsible for producing flagellin and type IV pili, are required for initial surface attachment, the first step in biofilm formation (O’Toole and Kolter, 1998). This and subsequent studies from other labs demonstrated that *ΔflgK* mutants have reduced biofilm formation (O’Toole and Kolter, 1998; Kang and Kirienko, 2017). Benyoussef et al. used a mutation in *flhD*, a flagellar regulatory gene, to study *E. coli* biofilm production and postulated that surface geometry might interact with flagellar motility proteins to impact biofilm production. The results suggest that more research on context-dependent interactions between the environment and motility genes would be beneficial.

The dos Santos Morais et al. study investigated biofilm formation, toxin production, and antibiotic sensitivity for three different strains of *Clostridioides difficile*. This anaerobic, spore-forming pathogen is well-known for causing serious nosocomial infections after antimicrobial-mediated disruption of the healthy microbiota, resulting in a variety of inflammatory conditions. The MLST Clade 2 strains examined in this study formed biofilms, which may increase the probability of recurring infections. ICC-45, a specific MLST2 strain, was resistant to vancomycin and metronidazole, raising its risk profile further.

Levipan et al. found that two different strains of *Piscirickettsia salmonis*, a Gram-negative pathogen that impacts aquaculture by causing severe infectious disease in fish, called piscirickettsiosis, can quickly form biofilms. To study the impact of this, the authors used transcriptome profiling to identify biofilm-associated genes, including those involved in polysaccharide biosynthesis and cell adhesion. Importantly, they observed no difference in virulence between planktonic and biofilm growth, suggesting that biofilm production in this species may represent a method of persisting in the aquatic environment, not the host.

Another area of active study in biofilm science is the connection of biofilms to the emergence of bacterial persisters. Persisters show surprising tolerance to antimicrobials, even those to which they are sensitive. This results in a pool of cells that can trigger recurrent infections (Lewis, 2007). Recent studies by Hobbs et al. linked increased persister numbers in *Staphylococcus aureus* to mutation of the *fumC* gene (Hobbs et al., 2021). Theis et al. examined the impact of *fumC* mutation on a murine catheter-associated biofilm model. Interestingly, while the infection of male mice resulted in similar CFUs of wild-type and *fumC* mutants, in female mice the difference in bacterial burden in tissues surrounding the catheter reached 2.5 logs. While that was insufficient for the statistical significance, these findings indicate a venue for more in-depth future research. Using a marker for persister cells, they identified increased numbers of persister cells and increased antimicrobial tolerance by cells with the marker in the biofilm. Using flow cytometry, they discovered that the cells were also less metabolically active. These findings support several hypotheses about persisters and biofilms.

A manuscript by Rowe et al. examined the role of alginate in resistance to phagocytosis and persistence in *P. aeruginosa*. Alginate production is typically associated with mucoid biofilms. Rowe et al. showed that alginate production decreased phagocytosis, possibly by blocking CD11b and CD14 receptors and inhibiting the activation of phagocytic signaling. Interestingly, however, exogenous alginate did not protect bacteria, suggesting that further research is warranted.

Since biofilms play an essential role in infection, the discovery of anti-biofilm agents is an area of active research. In this Research Topic, Board-Davies et al. present their findings on characterizing porphyrin-based XF drugs, a new class of antibacterials developed by Destiny Pharma plc. These molecules were shown to kill *S. aureus*, including in biofilms (Ooi et al., 2010). Board-Davies et al. extended these discoveries by examining the light-activated photodynamic properties of XF drugs on their anti-biofilm and bactericidal properties. Authors identified synergy between XF-73 or XF-70 and ertapenem in the treatment of *S. aureus*, and synergy with polymyxin B against *P. aeruginosa*, increasing their clinical promise.

In summary, there is increasing evidence that biofilms play a crucial role in infection of the host and growth in the environment. The papers in this Research Topic add to our understanding of how biofilms contribute to pathogenesis and virulence, which may reveal new ways to treat and prevent diseases and promote health.

## Author contributions

NK drafted the editorial. YD and NK revised the editorial. Both authors contributed to the article and approved the submitted version.
